# 3D bioprinting for the production of a perfusable vascularized model of a cancer niche

**DOI:** 10.3389/fbioe.2025.1484738

**Published:** 2025-01-29

**Authors:** Federico Maggiotto, Lorenzo Bova, Sara Micheli, Camilla Pozzer, Pina Fusco, Paolo Sgarbossa, Fabrizio Billi, Elisa Cimetta

**Affiliations:** ^1^ Department of Industrial Engineering (DII), University of Padua, Padova, Italy; ^2^ Fondazione Istituto di Ricerca Pediatrica Città della Speranza (IRP), Padova, Italy; ^3^ UCLA Department of Orthopaedic Surgery, David Geffen School of Medicine, Los Angeles, CA, United States

**Keywords:** 3D bioprinting, vascularization, perfusion, cancer niche, metastasis, neuroblastoma

## Abstract

The ever-growing need for improved *in vitro* models of human tissues to study both healthy and diseased states is advancing the use of techniques such as 3D Bioprinting. We here present our results on the development of a vascularized and perfusable 3D tumor mimic for studies of the early steps of Neuroblastoma metastatic spread. We used a multi-material and sacrificial bioprinting approach to fabricate vascularized 3D cell-laden structures and developed a customized perfusion system enabling maintenance of growth and viability of the constructs for up to 3 weeks. Cell phenotypes and densities in co-culture for both the bulk of the construct and the endothelialization of the vascular channels were optimized to better replicate *in vivo* conditions and ideally simulate tumor progression. We proved the formation of an endothelium layer lining the vascular channel after 14 days of perfused culture. Cells in the bulk of the construct reflected Neuroblastoma growth and its tendency to recruit endothelial cells contributing to neovascularization. We also collected preliminary evidence of Neuroblastoma cells migration towards the vascular compartment, recapitulating the first stages of metastatic dissemination.

## 1 Introduction

Three-dimensional (3D) cultures and cell-laden hydrogel constructs are now a standard in biomedical research, enabling the creation of more physiologically relevant models and providing specific mechanical cues to control cell fate and function (Moroni et al., 2018; Ouyang et al., 2020a; Zhang et al., 2019). Advances in 3D bioprinting combined with other additive manufacturing techniques are increasing the precision and fidelity of the shape of the tissue mimics, while also favoring the creation of patient-specific patches (Baker and Chen, 2012; Murphy and Atala, 2014). The ability to recapitulate components of the *in vivo* environment using 3D bioprinting is key for studies on the complexity of the tumor microenvironment (TME), which are strongly limited if conducted on 2D or scaffold-free 3D cultures (Erler and Weaver, 2009; Sánchez-Salazar et al., 2021; Fusco et al., 2019; Hoarau-Véchot et al., 2018). Bioprinted tumor models have been produced in a wide variety of simple structures ranging from discs, grids, fibers, mini organs, and custom shapes (Albritton and Miller, 2017; Langhans, 2018; Aazmi et al., 2022; Samadian et al., 2021; Kim and Kim, 2020). To achieve clinical relevance, tissue constructs must be more organized and significantly thicker than the few hundred micrometers where diffusion is sufficient to deliver nutrients to cells (Wang et al., 2021; Hinton et al., 2015; Gungor-Ozkerim et al., 2018; Zimmermann et al., 2006; Carrier et al., 1999; Juhas et al., 2014), thus requiring strategies to guarantee a constant provision of factors to all cells, including those in the bulk. Several sacrificial bioprinting techniques have been developed to create microchannels embedded in 3D-printed structures (Skylar-Scott et al., 2019; Richards et al., 2017; Kim et al., 2020; Kolesky et al., 2016). Examples are alternative print-casting, in which sacrificial materials such as Pluronic F-127 or carbohydrate glass (Ouyang et al., 2020b) are printed on a cast layer of cell-embedded matrix to shape a vascular network and multi-material bioprinting (Ouyang et al., 2020b), in which the vascularized construct is printed layer-by-layer alternating the use of two hydrogels and increasing the repeatability and consistency of the fabrication process. The sacrificial material is typically gelatin laden with endothelial cells, which adhere to the walls of the vascular channel after liquefaction of the sacrificial ink. However, the several operator-dependent procedures of the first approach and the use of gelatin as a sacrificial ink in the latter which limits the thickness of 3D constructs given the lack of mechanical strength still need to be optimized.

We here propose a manufacturing method for vascularized multi-cellular 3D constructs that combines the advantages of Pluronic F-127 as sacrificial ink and the fabrication speed of multi-material bioprinting. Thick vascularized constructs were printed using Gelatin Methacrylate (GelMA) as cell-laden matrix and Pluronic F-127 (PLU) as sacrificial ink (Alexandridis et al., 1995). GelMA was compatible with several cell types, including Neuroblastoma tumor cells (NB, SK-N-AS), human bone marrow-derived mesenchymal stem cells (hMSCs) and human umbilical vein endothelial cells (HUVECs). We also designed a customized perfusion system, enabling to maintain 3D constructs in culture for over 3 weeks. The ideal culture and co-culture conditions for both the bulk of the construct and the endothelialization of the vascular channel were determined assessing cell morphology and function. Our perfusable endothelialized vasculature integrated in a thick cancer niche will enable studies of cancer cell migration mimicking early-stage metastatic dissemination.

## 2 Materials and methods

### 2.1 GelMA synthesis

GelMA was synthesized in the laboratory following the protocol proposed by Shirahama et al. (2016). A 10% (w/v) solution of GelMA with 70% degree of functionalization (DoF, Supplementary Figure S1A) was prepared by initially dissolving a type A gelatin (∼300 bloom from porcine skin; Sigma-Aldrich) in 0.25 M carbonate-bicarbonate buffer (CB buffer) (sodium carbonate and anhydrous sodium carbonate in 1 × PBS; AppliChem) for 20 min at 40°C and constant stirring (800 rpm). The pH was adjusted to 9.2–9.4 by adding HCl (Hydrochloric acid 37%; Sigma-Aldrich). When the solution cleared, 50 µL of methacrylic anhydride (94%; Sigma-Aldrich) per Gram of gelatin were added dropwise; the methacrylation reaction was maintained for 2 h at 40°C and constant stirring (800 rpm). The reaction was quenched by adding HCl, bringing the pH to a physiological value of 7.4. GelMA was then collected, centrifuged (3,500 rpm, 5 min) and poured into dialysis membranes (14 kDa MWCO; Sigma-Aldrich). Dialysis lasts 5 days, keeping the membranes fully immersed in milli-Q water at 40°C with gentle and constant agitation (100–150 rpm); dialysis water was replaced at least three times per day. GelMA was filtered through a 0.22 µm filter and loaded into Falcons with 0.22 µm filter cap (50 mL bio-reaction tube; Celltreat) before being lyophilized for 7 days. Freeze-dried product can be stored at −20°C until use, keeping it isolated from moisture.

### 2.2 Bioinks preparation

The bioink we used as a cell-laden matrix was a solution of GelMA (8% w/v) and Irgacure 2959 (0.5% w/v) (Sigma-Aldrich) in 1 × PBS. The photoinitiator was dissolved in warm 1 × PBS (40°C) for 20 min with constant stirring (800 rpm), then the solution was filtered (0.22 µm filters) into a Falcon tube containing the lyophilized and weighted GelMA. The mixture was kept at 37°C and intermittently vortexed until GelMA completely dissolved. Before use, the ink was centrifuged to remove air bubbles (3,500 rpm, 5 min).

The sacrificial ink used to form the vascular network was a solution of 40% Pluronic F-127 (powder; Sigma-Aldrich) in cold (4°C) 1 × PBS. Mixing and intermittent vortexing led to a clear solution.

### 2.3 GelMA characterization

GelMA 8% was characterized according to mechanical and rheological tests described in Bova et al. (2022); of particular interest in this study are reported the evaluation of porosity and diffusion coefficient.

#### 2.3.1 Porosity

Porosity was studied by scanning electron microscopy (SEM; FEI Quanta 400 Scanning Electron Microscope) on 8% GelMA disks. GelMA samples were polymerized inside 6-mm diameter cylindrical PDMS molds following both manual deposition (standard casting) and dispensing via 3D bioprinter, and incubated in 1 × PBS for 24 h, then lyophilized and observed. During preliminary tests, no significant differences in porosity emerged between the different methods (data not shown), so we fabricated all additional disks using conventional casting. Quantification was performed with a custom Matlab function thresholding the image to highlight pores and divide them according to their size.

#### 2.3.2 Diffusion coefficient

The diffusion coefficient was studied by fluorescence recovery after photobleaching (FRAP) assays. Prior to confocal microscope acquisition, 6-mm diameter cylindrical samples of 8% GelMA were fabricated and immersed overnight in solutions of FITC-labeled dextrans of different molecular weights (4 kDa, 70 kDa, 250 kDa; Sigma-Aldrich). The half recovery time (*τ*_1/2_) characterizing the process (Hendrik Deschout et al., 2010) was estimated using a custom Matlab function reconstructing the intensity recovery curves from the averaged data measured within a selected ROI. The diffusion coefficient was then calculated according to the following Equation (Equation 1) (Hendrik Deschout et al., 2010), where *r* is the radius of the ROI:

$$D=\frac{2r^{2}}{\tau_{1/2}}$$ (1)

### 2.4 Printing trials

To optimize the Pluronic F127 concentration, the feasibility of printing vertical pillars was tested (Supplementary Figure S1B). Briefly, Pluronic 30, 40% and 50% were used to print pillars of different heights and monitoring their stability over 30 s. Pluronic 40% yielded the best results and was thus further characterized and used for all experiments. The filament was evaluated in terms of printing pressure and nozzle size (Supplementary Figure S1C), and printability was studied according to the method proposed by Ouyang et al. (Ouyang et al., 2016). We printed a test structure consisting of a 3 × 3 grid 2 × 2 mm in size, and microscope images were used to evaluate printability according to the formula in Equation 2 (Supplementary Figure S1D):

$$Printability\left(\mathrm{Pr}\right)=\frac{p^{2}}{16A}$$ (2)

The parameter Pr is 1 for a perfect rectangle, <1 for a rounded geometry, and >1 for jagged geometries.

### 2.5 Numerical simulations

We used COMSOL Multiphysics^®^ to simulate the flow of medium and the diffusion of oxygen in the vascularized 3D model. The “Free and Porous Media Flow” module relies on the Navier-Stokes equations to characterize flow in the free region (vascular channel) and the Forcheheimer-corrected Brinkman equations in the porous region (GelMA), yielding velocity and pressure fields. To analyze the concentration and transport of diluted species we used the “Transport of Diluted Species in Porous Media” module; the diluted species concentration was computed as the volume average concentration within the GelMA domain. The main parameters used in our models are reported in Table 1.

**TABLE 1:** Modeling parameters. Values of the main parameter used for the flow and diffusion simulations.

Parameter	Value	Unit of measurement
Density (GelMA)	1,150	kg/m^3^
Water content (GelMA)	1,058	kg/m^3^
Young’s modulus (GelMA)	8	kPa
Dynamic viscosity (GelMA)	100	Pa·s
Porosity (GelMA)	0.7	—
Permeability (GelMA) (Miri et al., 2018)	1·10^−7^	m^2^
Oxygen diffusivity	3·10^−9^	m^2^/s

### 2.6 Cell culture

SK-N-AS NB cells were cultured in Dulbecco’s modified Eagle medium (DMEM) supplemented with 10% FBS, 1% MEM and 1% P/S, renewing the medium every 3 days.

HUVECs were cultured in EGM-2 (Endothelial Cell Basal Medium 2) supplemented with SupplementPack (PromoCell) and used up to the 10th passage, renewing the medium every 3 days.

Human Mesenchymal Stem Cells (hMSC, ATCC 70022) were cultured in MesenCult MSC Basal Medium (Human) (STEMCELL) with 10% MesenCult™ MSC Stimulatory Supplement (Human) (STEMCELL), 1% Penicillin-Streptomycin, 1% Glutamine. hMSCs were used between passages 4 and 7.

### 2.7 Perfusion system and bioreactor design

The bioprinted construct was housed in a bioreactor, allowing connection to a perfusion circuit while maintaining sterile conditions. The device was designed with AutoCAD and 3D printed in PLA (Polylactic acid) (Prusa i3 MK3S+). Further details on each bioreactor element are discussed in the Results section (paragraph 3.4). Before use, the system was sterilized with 70% ethanol and exposed to UV light overnight.

### 2.8 Fabrication of the vascularized constructs

**FIGURE 1:**
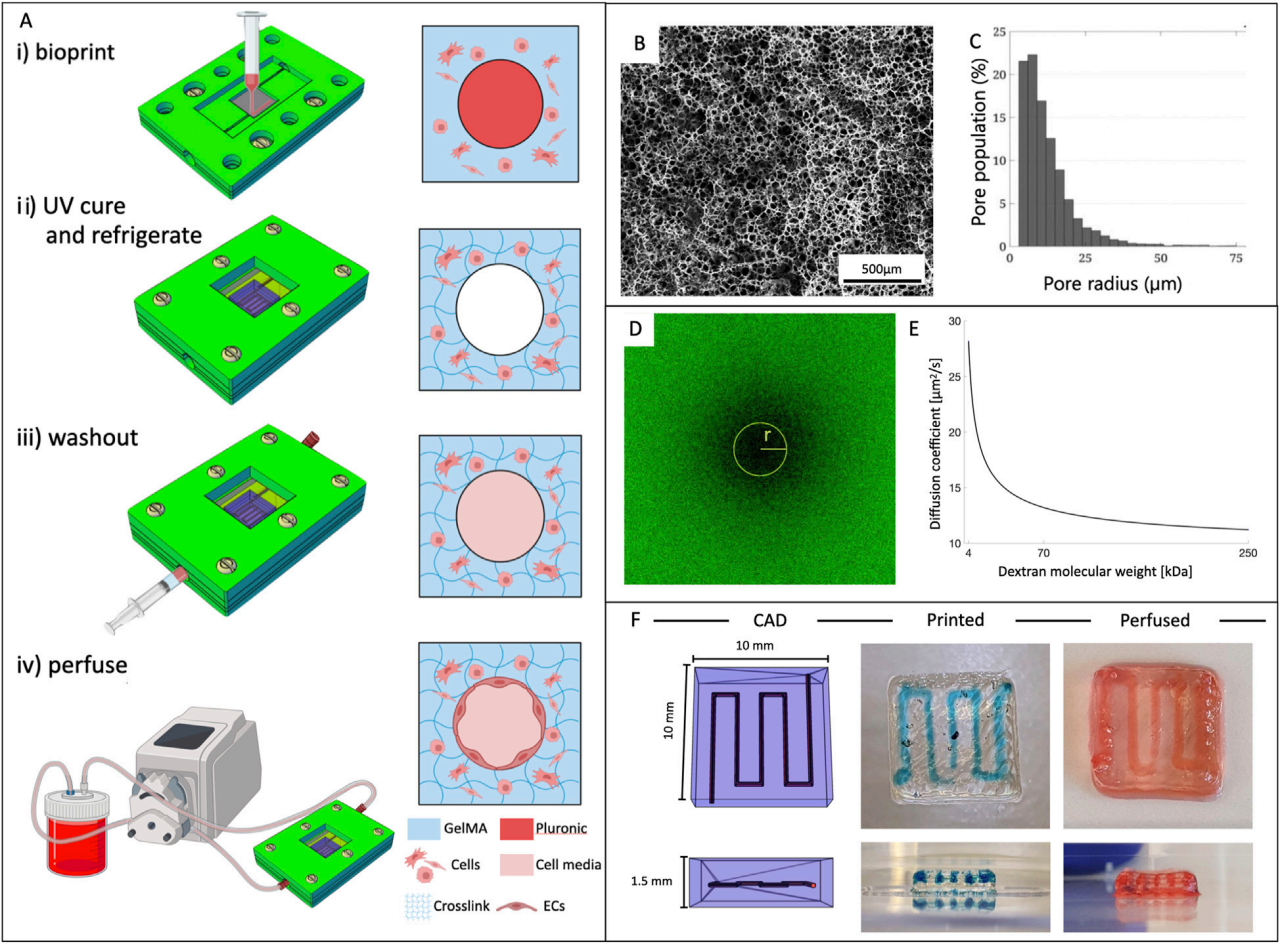
Vascularized constructs fabrication and bioink properties. **(A)** Outlined protocol for fabrication of vascularized 3D constructs. i) structure bioprinting inside the bioreactor; ii) UV exposure to crosslink GelMA and refrigeration for dissolving the sacrificial material (PLU); iii) washout of PLU by injecting cold fluid; iv) connection to the perfusion circuit. **(B)** Representative SEM image of the internal microstructure of 8% GelMA and **(C)** pore size distribution. **(D)** Confocal image of a bleached sample during FRAP testing and **(E)** diffusion coefficient of 8% GelMA as a function of molecular weight of dextrans. **(F)** From left to right: CAD model of the vascular channel geometry, bioprinted structure (PLU in blue), and after dye perfusion.

We fabricated all vascularized constructs using a BioX™ 3D bioprinter (CELLINK), featuring three independent print heads mounted on a three-axis system. This bioprinter offers precise temperature control for both the print bed and the print heads. Our fabrication process for creating thick vascularized constructs was based on the multi-material bioprinting method. In this approach, we sequentially co-printed two hydrogels following the specifications outlined in the G-code file. The two bioinks were loaded into 3 mL cartridges: GelMA, our primary material, was positioned into a temperature-controlled print head set at 23°C to ensure optimal extrusion, while PLU was left at room temperature. The bioinks were deposited within the bioreactor using 25G tapered and straight nozzles, respectively. A printing bed temperature of 22°C promoted the physical gel formation of GelMA. The printed structure was then exposed to UV light (365 nm, 90 s) to fully cross-link GelMA. Finally, we refrigerated the construct at 4°C for 5 min to liquefy Pluronic, which was then washed out by injecting cold 1 × PBS followed by culture media. This process resulted in the creation of hollow channels (Figure 1A).

For the development of cell-laden constructs, we used a cell-to-GelMA ratio of 1:25 to ensure that the hydrogel’s properties remained unaltered (Law et al., 2018). Optimized cell densities were as follows: 5·10^6^ SK-N-AS, and 1.5·10^6^ hMSCs per mL of hydrogel.

### 2.9 Experimental setup for perfusable constructs

**FIGURE 2:**
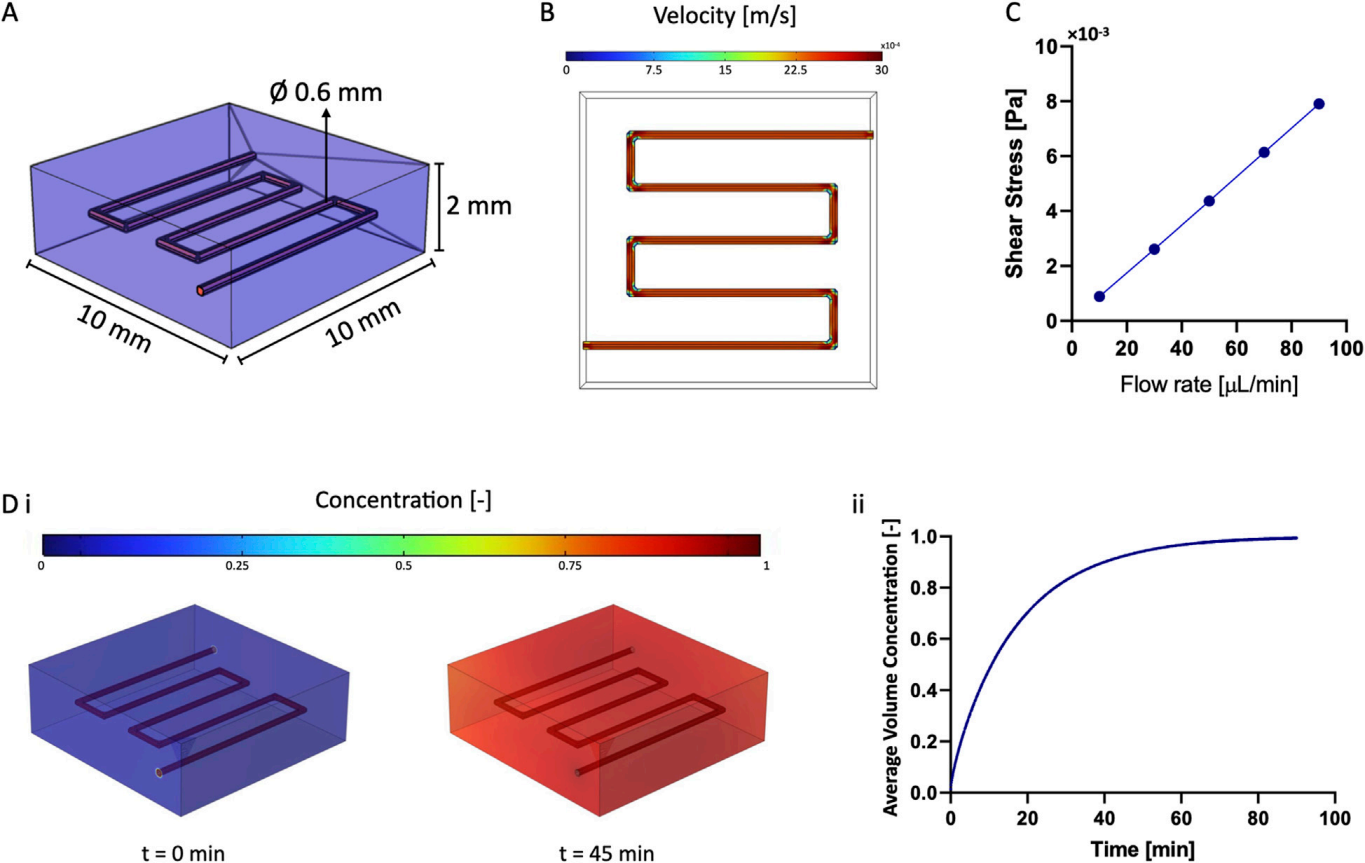
Numerical Simulations. **(A)** 3D model used for numerical simulations: the vascular channel was the free domain, while GelMA was the porous domain. **(B)** Flow pattern within the vascular channel. **(C)** Result of the parametric sweep to estimate the value of wall shear stress by varying the flow rate. **(D)** Diffusion of diluted species from the vascular channel to the surrounding GelMA: the target concentration was reached after approximately 45 min and was maintained over time, as shown in ii).

We established a continuous flow of cell medium through the vascular channel by connecting the construct to a perfusion circuit (Figure 2A). This circuit comprised a peristaltic pump (Dülabo PLP 380), a reservoir, and the bioreactor housing the construct. To connect the components, two silicone gas-permeable hoses with an inner diameter of 0.51 mm (Idex ISMATEC), were fitted onto the cartridge of the peristaltic pump head. These hoses were then connected to the ends of the vascular channel through two flexible-tip needles (22G, DRIFTON). The circuit was placed inside an incubator set at 37°C and 5% CO_2_. The peristaltic pump’s rotation speed was adjusted to achieve a flow rate of 10 μL/min for the first 2 days of culture, then it was increased to 30 μL/min for the entire duration of the experiment.

### 2.10 Endothelialization of vascular network

We optimized the endothelialization of the vascular channels using a post-seeding method after completion of the printing procedures. Once Pluronic F127 had been thoroughly removed from the channels as outlined in Section 2.8, we introduced HUVECs and hMSCs into the construct at a combined density of 10·10^6^ cells/mL_medium_, with a ratio of 70% HUVECs to 30% hMSCs. hMSCs were added for their proven role in promoting and facilitating HUVECs proliferation and the formation of the endothelial barrier (Lü et al., 2018; Kang et al., 2013). The structure was incubated at 37°C for 4 h, gently flipping it every 30 min for 4 h. After this initial incubation period, we gently removed any unattached cells with warm medium. Following successful endothelialization, the structure could be integrated into the perfusion circuit and maintained flowing HUVECs medium.

### 2.11 Cell viability assays

Cell viability was evaluated through a LIVE/DEAD assay by staining the samples with a solution of calcein-AM (“live”; 1:1,000), propidium-iodide (“dead”; 1:250), and HOECHST 33342 (1:500), marking all nuclei.

After quartering the structures and performing three washing cycles in 1 × PBS (5 min each), the samples were immersed in the staining solution for 45 min and incubated at 37°C. The samples were then washed with 1 × PBS (three washing cycles, 5 min each) before collecting images under a fluorescence microscope (Invitrogen EVOS FL).

### 2.12 Fixing and staining

The vascularized structures were fixed and stained for confocal imaging. The constructs were first washed with 1 × PBS (3 cycles, 5 min each) and fixed in paraformaldehyde (PFA, 4% v/v) at 4°C; for 4 h. After an additional washing with 1 × PBS (3 cycles, 5 min each), they were permeabilized using PBS-T (Triton X-100 0.1% in 1 × PBS) for 30 min. F-actin (1:250, 45 min) and DAPI (1:500, 15 min) staining were preceded by a rinsing step with 1 × PBS (3 cycles, 5 min each). Confocal microscopy was performed on a ZEISS LSM 800, using spectral lasers at 561 and 455 nm wavelength.

### 2.13 Immunostaining

First, the samples were washed with 1 × PBS and fixed in 4% PFA for 4 h at 4°C as described in 2.12. After PBS-T washes (Triton X-100 0.1% in 1 × PBS), the samples were immersed overnight in a blocking solution consisting of BSA 5% (Bovine Serum Albumin, from Sigma Aldrich) in PBS-T. The primary antibodies diluted in blocking solution were added to the structures and incubated for 2 days (E-Cadherin 1:50 from GeneTex; Vimentin 1:50 from GeneTex; CD31 1:100 from ProteinTech). Samples were then washed with PBS-T and the secondary antibodies diluted in blocking solution were added and incubated overnight.

### 2.14 HUVECs sprouting

The angiogenic behavior of HUVECs was studied by analyzing the obtained fluorescence images with ImageJ’s “Angiogenesis analyzer” tool (Carpentier et al., 2020). The plug-in detects and analyzes the pseudo vascular organization that endothelial cells, under appropriate culture conditions, could form. Early stages typically involve the formation of branching structures that can mimic capillary formation *in vitro*, while further differentiation can lead to a more mature mesh of different sizes (Montesano et al., 2024). The Angiogenesis Analyzer is thus a tool to quantify images from the Endothelial Tube Formation Assay (ETFA) experiment by extracting characteristic network information.

### 2.15 Vascular permeability measurements

To assess the barrier function of the bioprinted channel, we measured the vascular permeability by adapting the method proposed by Kolesky et al. (2016). Briefly, FITC-labeled 70 kDa dextrans were perfused through the channel, either in the presence or absence of endothelial cells, at a flow rate corresponding to the nominal operating rate during culture. The perfusion lasted for 30 min, with images captured every 30 s in regions surrounding the vascular channel, subsequently analyzed using ImageJ. The vascular permeability value was then calculated as reported in Equation 3:

$$P=\frac{1}{I_{0}-I_{b}}\left(\frac{I_{t}-I_{0}}{t}\right)\frac{d}{4}$$ (3)

where *P* represents the vascular permeability value [cm/s], *I*_*0*_ is the intensity within the ROI at the initial time point, *I*_*t*_ is the intensity at time *t*, *I*_*b*_ is the background intensity (prior to dextran perfusion), and *d* is the average diameter of the vascular channel (n = 3).

### 2.16 Statistical analysis

All measurements reported were taken from independent samples. Data were analyzed in Excel (Microsoft) and plotted in Prism (GraphPad) or Biorender. Data are shown as median±SD or mean ± SD for a given number of biological replicates (n ≥ 3). Significant differences were defined as *p < 0.05, **p < 0.01, ***p < 0.005, ****p < 0.001, unless otherwise noted. Differences between the experimental groups were analyzed by unpaired, two-tailed Student’s t-test, or one-way ANOVA.

## 3 Results and discussion

### 3.1 Bioinks preparation and characterization

Our multi-material approach is based on the use of gelatin methacrylate (GelMA) for the bulk of the construct and Pluronic F-127 as a sacrificial ink to build the perfusable channels.

GelMA is chemically synthesized by functionalizing gelatin with methacrylic anhydride to enable photo crosslinking. The key parameter to monitor for GelMA synthesis is the degree of functionalization (DoF, or degree of substitution) representing the percentage of lysins functionalized with methacrylate groups. We determined the DoF for every synthetized batch using H-NMR analysis, measuring a consistent value of 70% ± 10% (Supplementary Figure S1A).

We chose 8% GelMA based on our previous studies and for its ideal mechanical and rheological properties and printability (Bova et al., 2022), performing additional porosity and diffusion coefficient evaluations.

Porosity was studied using scanning electron microscopy (SEM) showing that GelMA was characterized by a tight mesh with pore radiuses in the 5–14 µm range (Figures 1B, C).

The diffusion coefficient was studied through Fluorescence Recovery After Photobleaching (FRAP) using dextrans of different molecular weights (4, 70, 250 kDa) to define the diffusion coefficient for a broader range of molecules. Results in Figures 1D, E confirm that the diffusion coefficient decreased with increasing molecular weight following a logarithmic trend (D_4kDa_ = 28.2 ± 2.9 μm^2^/s, D_70kDa_ = 13.2 ± 3.3 μm^2^/s, D_250kDa_ = 11.2 ± 1.8 μm^2^/s).

### 3.2 Printing of bioinks

Both materials underwent rigorous testing across various conditions to optimize the key parameters affecting the 3D printing process. GelMA had exceptional printability (*Pr* = 0.93 ± 0.01) with 25G tapered needles and a 5 mm/s printing speed at a temperature of 23°C. Pluronic exhibited excellent definition for a wider range of parameters, particularly when printing complex geometries and free-standing 3D structures. We evaluated three concentrations, and soon excluded the lower at 30% as excessively liquid. Both the 40% and 50% concentrations yielded positive results. Ultimately, we selected Pluronic 40% for its superior long-term stability and printability. For Pluronic 40%, the calculated *Pr* values when printed with 25G and 27G nozzles were 0.95 ± 0.02 and 0.96 ± 0.03, respectively, indicating excellent printability with no statistically significant difference between the two nozzle sizes. After careful evaluation of filament size and printing pressures, the 25G nozzle was chosen to balance the obtainment of a relatively larger filament size with the advantage of requiring the lower pressure range of 300–350 kPa. This protocol consistently yielded thick perfusable 3D constructs (Figure 1F).

### 3.3 Numerical simulations

We determined and validated the efficiency of medium perfusion within the vascular channel using COMSOL Multiphysics. Predicting the material behavior is crucial for understanding the convection and diffusion mechanisms that dictate cell viability and physiologic activity within the model (Figure 2A).

The computational model aimed at determining the flow pattern within the channel and the characteristic time for diffusion of diluted species from the vascular channel to the surrounding matrix. We first confirmed that the flow within the channel reached the steady state almost instantly (Figure 2B). We then examined the effect of varying the flow rate (from 10 μL/min to 90 μL/min) to determine the shear stress at the walls of the vascular channel. From this parametric sweep, we evaluated that the wall shear stress increased linearly from 0.89 10^−3^ to 7.90 10^−3^ Pa, allowing to estimate the force acting on the endothelial cells lining the channel walls (Figure 2C). Our nominal operating flow rate of 30 μL/min was consistent with literature values (Kolesky et al., 2016) although it resulted in far lower shears than those reported for *in-vivo* studies (Ballermann et al., 1998).

Additionally, we simulated the characteristic diffusion time of diluted substances to reach a target concentration within the matrix surrounding the vascular channel (Figure 2D). Modeling results indicated that the target value was reached throughout the 3D constructs after approximately 45 min and was maintained over time. This confirmed that all cells in culture would receive a homogeneous and physiologic supply of nutrients and oxygen within less than an hour from the initiation of perfusion.

### 3.4 Bioreactor and perfusion circuit

**FIGURE 3:**
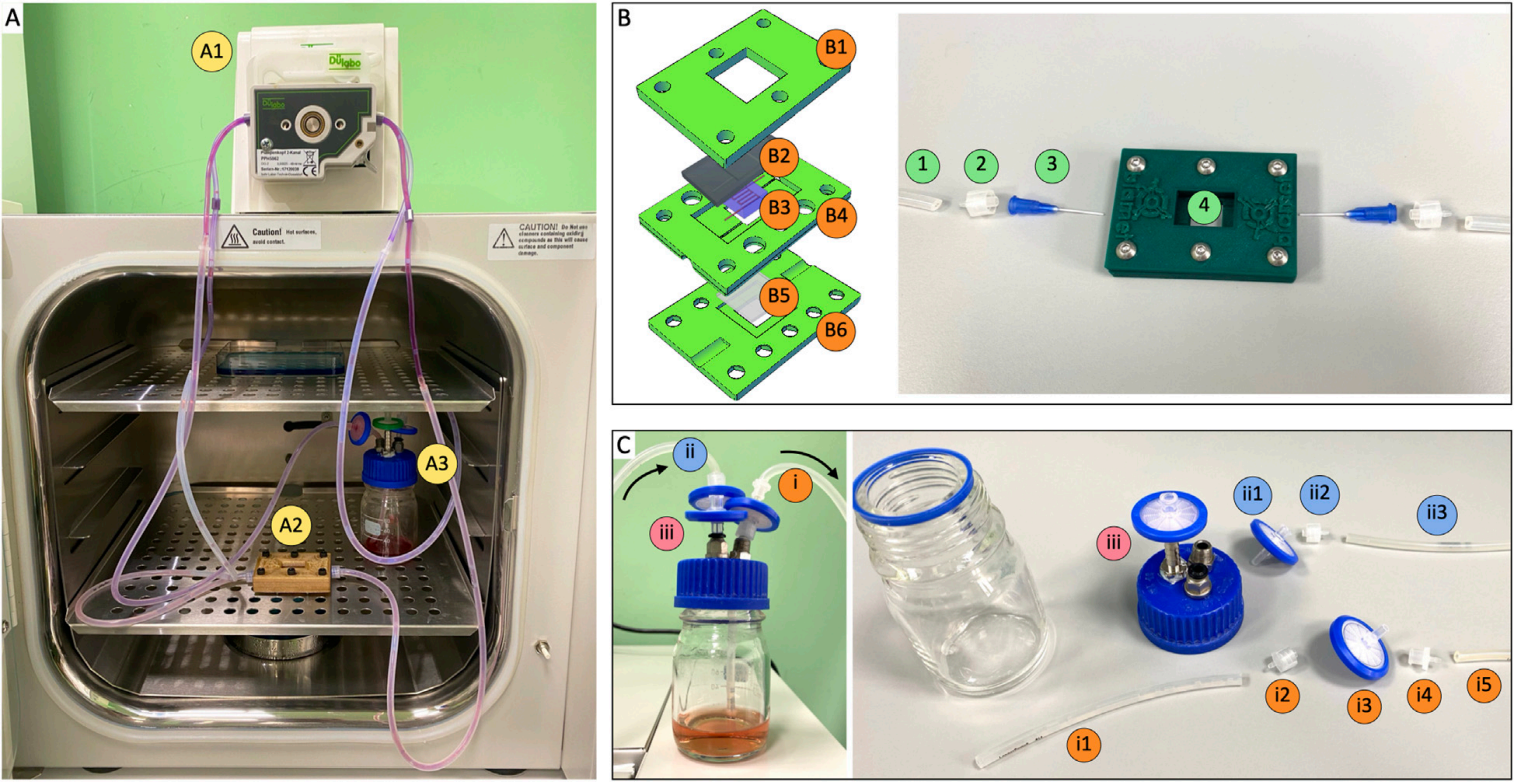
Perfusion circuit components. **(A)** Perfusion circuit composed by A1) peristaltic pump, A2) bioreactor and A3) cell medium reservoir. **(B)** Left: bioreactor design. B1) top blocking frame, B2) PDMS layer, B3) vascularized construct, B4) bioreactor body, B5) coverslip with PDMS coating, B6) bottom supporting plate. Right: connection to perfusion circuit. 1) silicone hose, 2) male luer lock, 3) plastic flexible tube tip 22G, 4) vascularized construct within bioreactor. **(C)** Reservoir configuration. I) suction section: i1. Silicone hose, i2. Male luer lock, i3. 0.22 µm filter, i4. Female luer lock, i5. Silicone hose. Ii) pouring section: ii1. 0.22 µm filter, ii2. Male luer lock, ii3. Silicone hose. Iii) 0.22 µm air filter. Inner diameter of all silicone hoses was 0.51 mm.

The experimental set up enabled prolonged culture of cellularized constructs under continuous perfusion (Figure 3A). We designed and fabricated a customized bioreactor composed of the following elements (Figure 3B): a top blocking frame, which securely sealed a PDMS membrane; the mid bioreactor body, consisting of a layer designed to house a coverslip and ensure precise placement of the inlet/outlet ports; and a bottom supporting plate. This assembly allowed us to print and crosslink the vascularized structure directly within the bioreactor, effectively isolating it from the external environment through the PDMS layer. A significant anticipated challenge was the proper insertion and secure positioning of needles within the channels to prevent leakage and protect the construct during critical steps such as Pluronic washout, cell seeding, and perfusion. To address it, we incorporated insertion holes into the sides of the bioreactor, sized to precisely accommodate the needles. This design enabled accurate positioning of the needles, ensuring they reached the extremities of the printed vascular channel. The assembled device was then integrated into the perfusion circuit, comprising a peristaltic pump and a medium reservoir (Figure 3C). We positioned a filtration system both before the medium inlet into the vascular channel and at the exit before recirculation into the reservoir, ensuring sterility throughout the perfusion process. All tubing had an internal diameter of 0.51 mm. The perfusion system requires two channels of a peristaltic pump; with four or more channels available, multiple devices can be simultaneously run in parallelized experimental campaigns.

### 3.5 Cell viability

**FIGURE 4:**
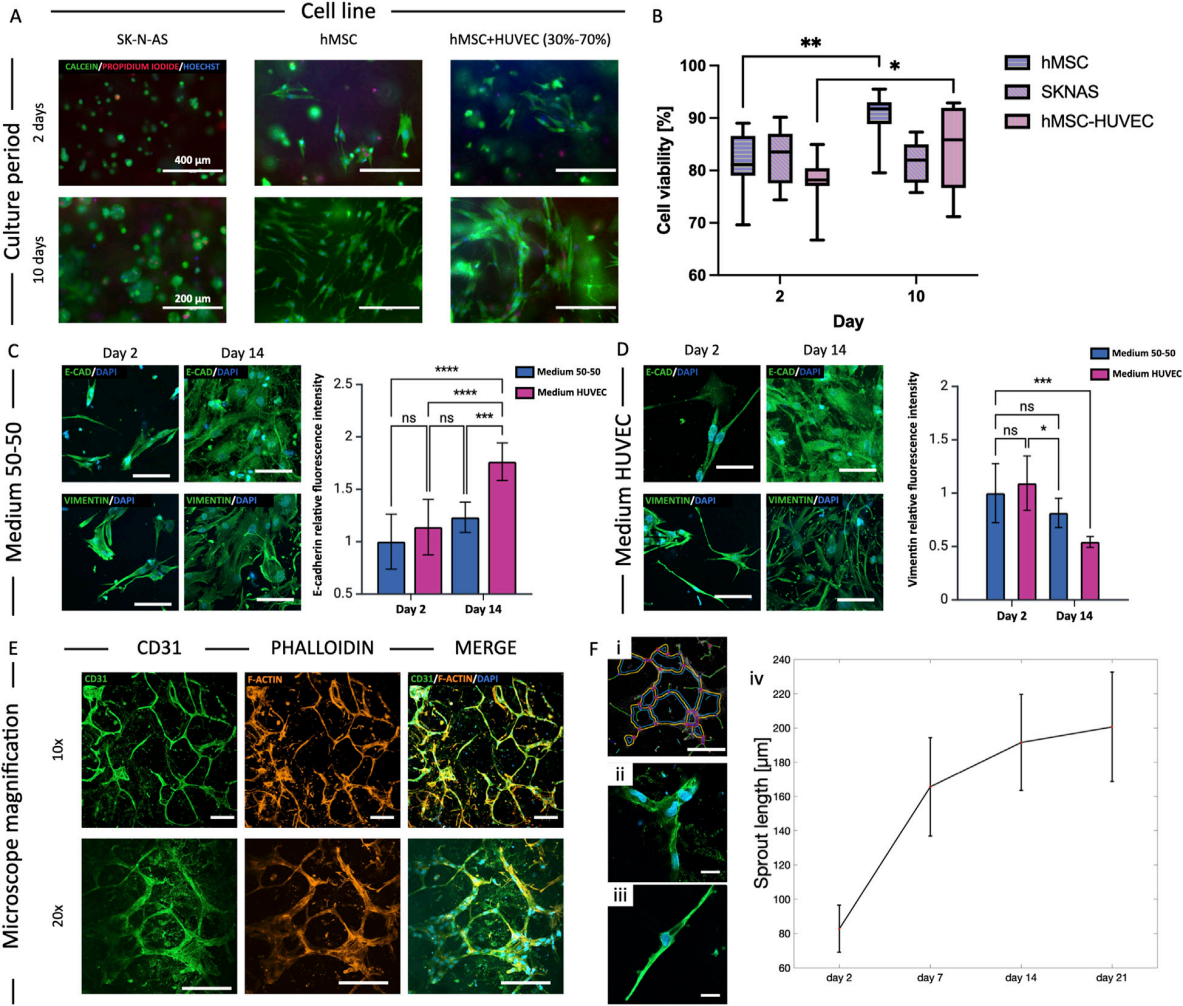
Cell viability and morphology. **(A)** Cell viability assay for the three cell cultures (SK-N-AS, hMSC and HUVEC-hMSC 70%–30%) in 8% GelMA: cytoplasm of live cells stained green (Calcein-AM), dead cells nuclei red (Propidium-iodide), and all nuclei in blue (HOECHST). **(B)** Quantification of cell viability at day 2 and 10 of culture (*p < 0.05, **p < 0.01, median±SD, n ≥ 3). **(C, D)** Study of the effect of medium composition on cell morphology and function of HUVEC-hMSC co-cultures in 8% GelMA: HUVEC medium promoted endothelial differentiation of hMSCs through increased expression of E-Cadherin and reduced Vimentin between day 2 and 14 of culture (***p < 0.001, mean ± SD, n = 3). Scalebar = 20 µm. **(E)** Cell morphology study of HUVEC-hMSC co-culture in 8% GelMA at day 14: spontaneous microvasculature formation with CD31 in green, actin filaments (phalloidin) in red and nuclei (DAPI) in blue. Scalebar = 50 µm. **(F)** Quantification of the sprout length using ImageJ plug-in “Angiogenesis Analyzer”. (*p < 0.05, **p < 0.01).

**FIGURE 5:**
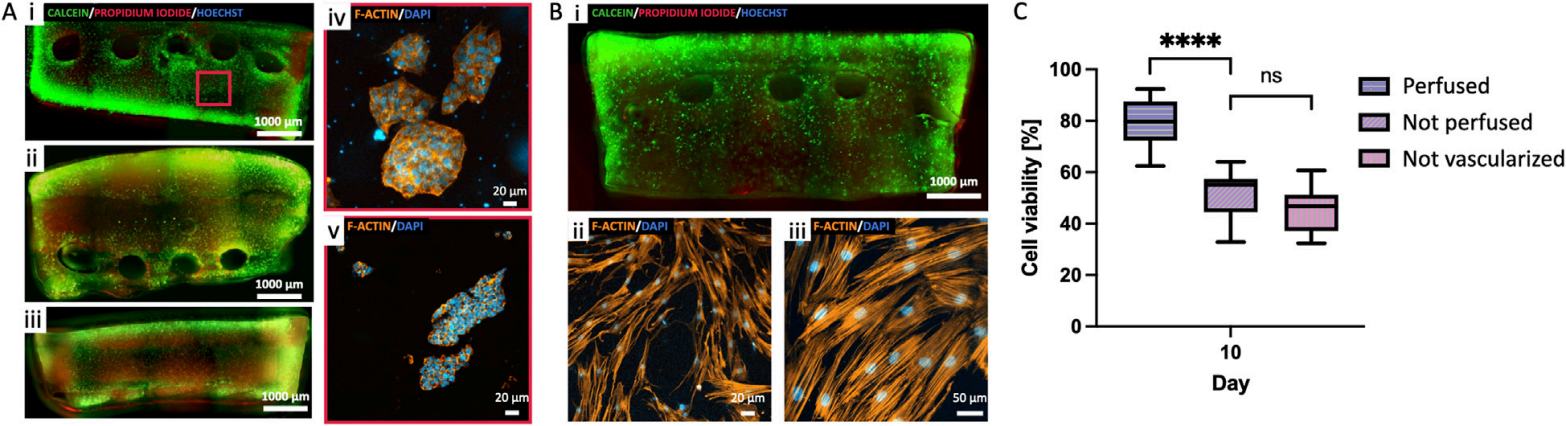
Perfusion of cell-laden constructs. **(A)** 3D bioprinted model of Neuroblastoma: comparison of i) vascularized perfused, ii) vascularized not perfused and iii) not vascularized not perfused. iv-v) Details of NB cells clusters formation in perfused samples at day 15. **(B)** 3D bioprinted model of hMSC: i) whole viable construct, and ii-iii) representative details of cell morphology in the perfused samples at day 15. **(C)** Quantification of cell viability (Calcein-AM-labeled green cell, and Propidium-iodide-labeled dead cells) in the perfused, not perfused and not vascularized NB constructs at day 15 (median±SD, n = 3). (****p < 0.001). Scalebars as indicated.

LIVE/DEAD assays were performed at day 2 and 10 on 3D GelMA constructs obtained casting 100 µL of GelMA-cells bioinks into a 10 mm, ∼1 mm high cylindrical PDMS mold. We assessed cell viability of SK-N-AS, hMSCs and HUVEC-hMSC co-culture (70%–30%) using calcein-AM, propidium-iodide, and HOECHST 3342 to stain live, dead cells, and all nuclei, respectively. The viability of SK-N-AS cells was high and stable over time, with aggregates that could grow up to 200 µm by day 10. hMSCs and HUVEC-hMSC co-culture resulted in increasing cell viabilities at the tested timepoints, possibly correlating with sustained proliferation over time (Figures 4A, B). Additional viability results for bioprinted models are detailed in Section 3.7 and reported in Figure 5. These data were consistent with our previous findings (Bova et al., 2022) measuring similar viabilities for casting and extrusion bioprinting and proving that the latter was not detrimental to cell survival.

### 3.6 Vasculogenesis and cell morphology

To address potential effects of medium composition on HUVEC-hMSC co-cultures in forming the endothelial barrier, we studied two formulations at different timepoints (day 2, 7 and 14): medium 50% HUVEC – 50% hMSC, and 100% medium HUVEC. Vimentin and E-Cadherin are known markers for mesenchymal-endothelial transition (MendoT), so we qualitatively analyzed their expression as an indicator of phenotypic changes (Ubil et al., 2014). While no significant differences could be detected when using a blended medium, the results show an increase in the expression of E-Cadherin (p < 0.001, n ≥ 3) and a decrease in Vimentin (p < 0.001, n ≥ 3) at day 14 when using 100% HUVEC medium (Figures 4C, D). The expression of the markers was qualitatively analyzed based on the average fluorescence intensity within each individual image, normalized to the number of cells. The data are presented as variations relative to the day 2 samples in 50–50 medium condition, which were considered the reference samples. Given the indication that HUVEC medium could stimulate hMSCs differentiation towards endothelial cells, we chose it for all co-culture experiments. We then studied the formation of closed structures and the sprout length of co-cultures using ImageJ’s “Angiogenesis Analyzer” plug-in. We observed the formation of organized meshes with multiple connections between cell branch endings and measured sprout lengths that increased steeply between days 2 and 7, and then more gradually until day 21 (Figures 4E, F).

### 3.7 Perfusion of cell-laden constructs

Constructs were formed embedding SK-N-AS NB cells and hMSC in 8% GelMA at cell densities of 5·10^6^ cells/mL and 1.5·10^6^ cells/mL, respectively. Once the structure was fabricated and the Pluronic F127 removed from the vascular channel, the cellularized construct was connected to the perfusion circuit by setting a continuous medium flow rate of 10 μL/min for the first 2 days of culture, then increasing it to 30 μL/min. Cell viability and morphology were assessed at day 7 and 15 through viability assays and staining. We compared vascularized and perfused constructs to two controls: one vascularized not perfused and one not vascularized not perfused. The results in Figure 5A demonstrate how perfused samples had significantly higher cell viabilities than not perfused and not vascularized samples, proving the importance of a perfused vascular system in providing nutrients to cells in the bulk of the constructs.

### 3.8 Endothelialization of the vascular channel

**FIGURE 6:**
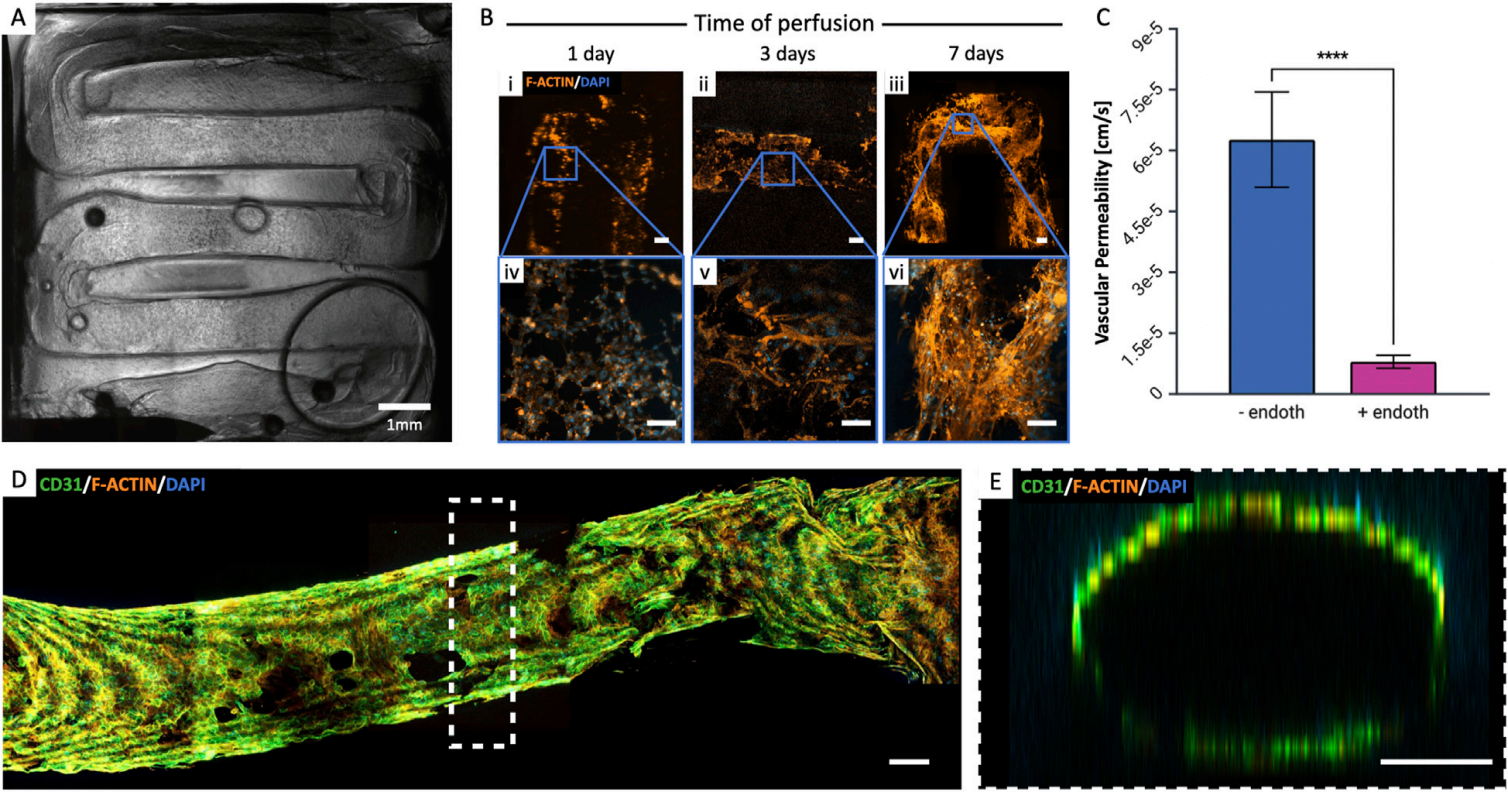
Endothelialization of the vascular channel. **(A)** Post-seeding view of the entire vascular channel (HUVEC-hMSC co-culture 70%–30%, 10·10^6^ cells/mL). **(B)** Formation of the endothelial barrier over time: at day 1 (i, iv), cells are still rounded, then appear to stretch (ii, v) and form a dense network as early as day 7 (iii, vi). Scalebar = 200 µm. **(C)** Vascular permeability was measured in the presence and absence of endothelium to validate the endothelialized channel’s functionality. The presence of endothelium significantly reduced permeability (***p < 0.001, n = 3), indicating its critical role in maintaining vascular barrier integrity. **(D)** Complete endothelium formation at day 14 of perfusion and **(E)** lumen formation. Scalebar = 200 µm.

We co-cultured HUVECs and hMSCs in a ratio of 70:30% to replicate the endothelial barrier, effectively separating the flow of medium from the surrounding matrix. The total cell density seeded in HUVEC medium was set at 10·10^6^ cells/mL. Following seeding, the structure underwent periodic flipping every 30 min for a total of 4 h. This action served two purposes: it prevented uneven gravity-driven cell adhesion and supported the formation of a uniform monolayer along the channel walls (Figure 6A). The constructs were then integrated and connected to the perfusion circuit, ensuring a consistent supply of medium to the cells and the application of shear forces. As in viability studies, the flow rate was initially set at 10 μL/min for the first 2 days of culture and then increased to 30 μL/min. This protocol optimization allowed cells sufficient time to adhere to the channel surfaces before the application of shear stress, which is necessary for aligning them in the direction of flow. Sample analyses were conducted at various time points to assess endothelial monolayer formation (specifically: on days 1, 3, 7, and 14, Figure 6B). To further validate the functionality of the endothelialized channel, we measured vascular permeability in the presence and absence of endothelium by perfusing FITC-labeled dextrans. The results, shown in Figure 6C, demonstrate that the presence of the endothelium significantly reduces vascular permeability (p < 0.001, n ≥ 3). This finding suggests that the endothelial layer plays a critical role in maintaining the integrity of the vascular barrier, as it effectively limits the passage of substances through the vessel walls. Cells organized over time, forming a dense network as early as day 7. By day 14, they had completely covered the vascular channel surface, and formed a fully developed lumen (Figures 6D, E). We also highlight the presence of image acquisition-related optical artifacts, which result in lower intensities for the innermost tissue layers compared to those closer to the microscope’s focal plane, and are thus not related to different cellularization densities.

### 3.9 Endothelialized neuroblastoma niche

**FIGURE 7:**
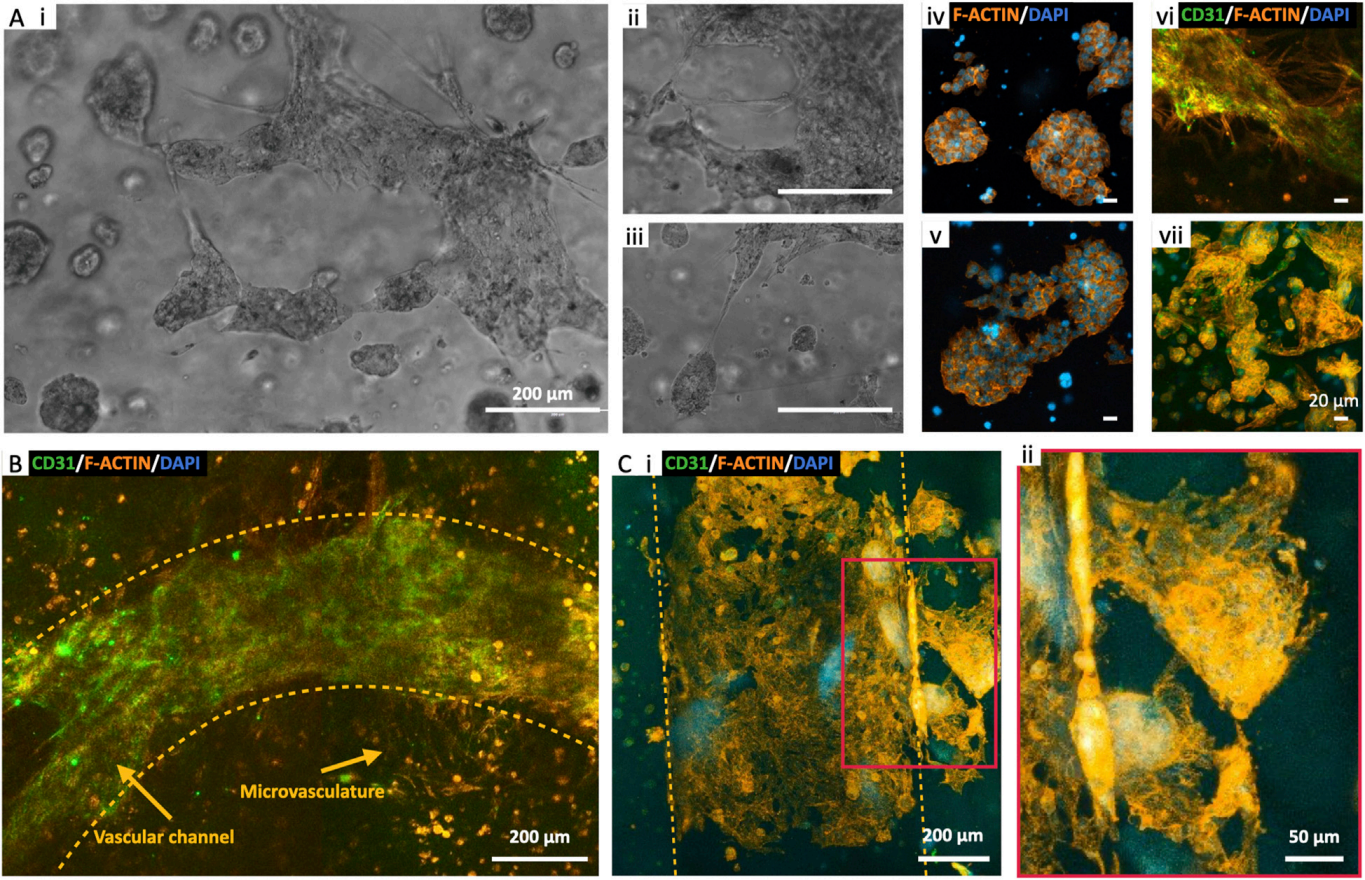
Endothelialized Neuroblastoma niche. **(A)** Perfused bioprinted 3D model of SK-N-AS-HUVEC co-culture in 8% GelMA: microvasculature formation in proximity of SK-N-AS aggregates at day 10 (i–iii) and day 21 of perfusion (iv–vii). **(B)** Endothelial barrier formation and sprouting toward the bulk of the 3D structure at day 14 of perfusion. **(C)** Microvasculature sprouting from the vascular channel and surrounding aggregates of SK-N-AS at day 21 of perfusion.

To enhance the physiological relevance of our findings, we integrated endothelialization of vascular channels with the perfusion of cellularized constructs. This approach aimed to create a more faithful representation of the capillary circulation within a tissue niche, acknowledging that the absence of an endothelial barrier in earlier experiments simplified the *in vivo* mechanisms. For these experiments, we optimized cell densities as follows: 5·10^6^ SK-N-AS/mL_GelMA_ and 1·10^6^ HUVEC/mL_GelMA_ for the bulk of the structure, and 10·10^6^ cells/mL_medium_ 70% HUVEC-30% hMSC for the channel endothelialization. Co-culturing NB cells and HUVECs served the purpose of promoting spontaneous microvasculature formation within the bulk of the construct which, upon maturation, are predicted to further improve nutrient and oxygen transport (Debbi et al., 2022). We maintained these multi-cell constructs under perfusion conditions for up to 21 days, using a mixed formulation of 50% SK-N-AS and 50% HUVEC medium (Jaganathan et al., 2014; Horie et al., 2015; Villasante et al., 2017). Microscope observations provided valuable insights, revealing the formation of SK-N-AS aggregates (>500 µm) within the bulk of the structure as early as at day 10. These aggregates could potentially act as attractants, further drawing microvessels from the surrounding tissue (Figure 7A i–iii). This process evolved throughout the culture period, more accurately mimicking the complex mechanisms of tumor angiogenesis and metastasis, with structures maturing by day 21 (Figure 7A iv–vii). Furthermore, the development of the vascular channel involved the growth of microvasculature branching from the primary channel (Figure 7B). Finally, as shown in Figure 7C, SK-N-AS aggregates located in close proximity to the vascular channel appeared to connect to the vascular compartment via sprouting protrusions, effectively recapitulating the initial stages of metastatic migration.

## 4 Conclusion

In this study, we proposed a comprehensive strategy for fabricating vascularized cell-laden constructs using advanced multi-material 3D bioprinting techniques. Our primary objective was to establish a vascularized Neuroblastoma model that could faithfully replicate and facilitate the investigation of the complex mechanisms driving the early stages of tumor metastatic dissemination.

The first and key step in any 3D bioprinting approach is the careful selection of a bioink. This material must possess properties allowing for precise printing and polymerization, ensuring the stability of the resulting constructs. In our case, the bioink also had to exhibit characteristics conducive to cell adhesion, be characterized by stiffness and elasticity to support proper cell proliferation, migration, and differentiation during the post-printing maturation process. Our material of choice, gelatin methacrylate (GelMA) at an 8% concentration (w/v), met these criteria. At this concentration, GelMA exhibited low viscosity, enabling extrusion at low pressures (∼50 kPa) while maintaining excellent printing precision. To create an environment conducive to the prolonged dynamic culture of bioprinted structures, we integrated our construct within a perfusion apparatus and a customized bioreactor. This unit also facilitated proper perfusion, ensuring precise positioning of the inlet and outlet needles throughout the entire production and operational phases.

Our experimental setup proved highly efficient in recreating a stable and suitable culture environment for bioprinted constructs, even during long-term cultures for up to 3 weeks. Additionally, we demonstrated our ability to recreate an endothelial barrier that effectively separated the culture medium from the cells within the construct. This was achieved through a co-culture of endothelial (HUVECs) and mesenchymal stem cells (hMSCs) at a concentration of 10·10^6^ cells/mL, with a composition of 70% HUVECs and 30% hMSCs. Monitoring the formation of the endothelium involved evaluating the lining of the vascular channel with cells, ultimately confirming the development of a complete cell monolayer after 14 days of perfusion. Moreover, by recreating endothelialized channels within a SK-N-AS NB cells laden construct, we highlighted the formation of elongations that connected clusters of NB cells to larger vessels, offering valuable insights into the initial stages of metastatic migration.

Moving forward, our research will further study the mechanisms of metastasis, with the developed model enabling to investigate the migration of primary tumor cells toward healthy tissues by connecting a tumor niche and a target tissue via an endothelialized channel. This setup will allow us to closely monitor cell behavior and key phenomena related to intravasation and extravasation over time. The ability to accurately recreate the endothelium is pivotal for constructing a physiologically and biologically relevant model that holds great promise for advancing our understanding of metastasis.

## Data Availability

The datasets presented in this study can be found in online repositories. The names of the repository/repositories and accession number(s) can be found below: https://researchdata.cab.unipd.it/909/.

## Supplementary material

SUPPLEMENTARY FIGURE S1 
**(A)** H-NMR graph confirming a GelMA DoF of 70% ± 10%. **(B)** Printing vertical pillars with Pluronic: the selected concentration is 40% as it allows for more stable vertical pillars over time. **(C)** Filament size of Pluronic 40% printed at different pressures with two printing nozzles. **(D)** Bioprinted structure to evaluate the printability parameter (Pr) of biomaterials.
