# Supplementary figures and images for: 3D bioprinting for the production of a perfusable vascularized model of a cancer niche
Source: Front Bioeng Biotechnol. 2025 Jan 29;13:1484738. doi: 10.3389/fbioe.2025.1484738 (PMC11841441; doi:10.3389/fbioe.2025.1484738)

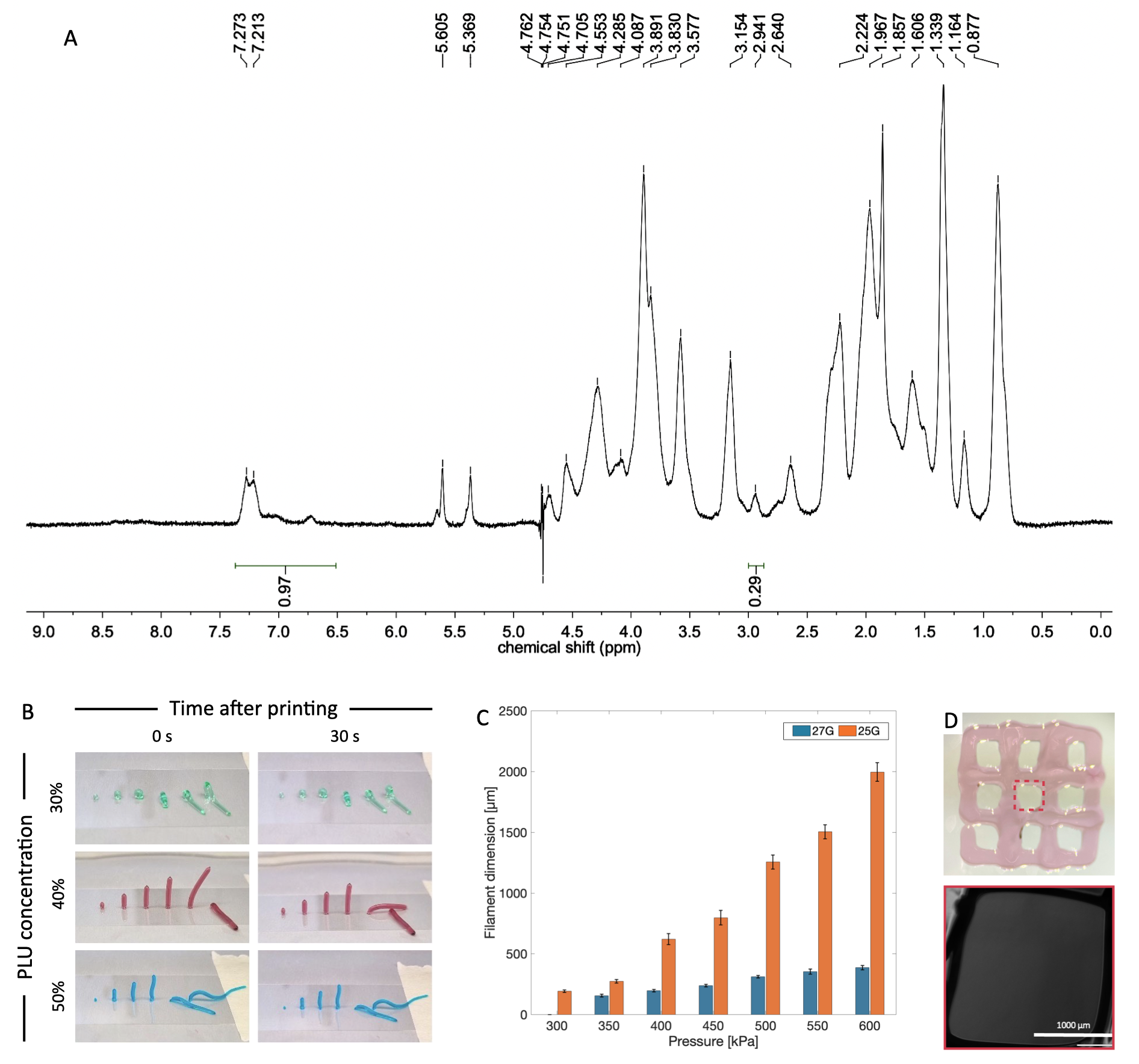

Supplement: Supplementary file 1 [file Image1.tiff]
